# Analyzing Twitter Data to Evaluate People’s Attitudes towards Public Health Policies and Events in the Era of COVID-19

**DOI:** 10.3390/ijerph18126272

**Published:** 2021-06-10

**Authors:** Meng Hsiu Tsai, Yingfeng Wang

**Affiliations:** Department of Computer Science and Engineering, University of Tennessee at Chattanooga, Chattanooga, TN 37403, USA; wmf223@mocs.utc.edu

**Keywords:** sentiment analysis, COVID-19, social media

## Abstract

Policymakers and relevant public health authorities can analyze people’s attitudes towards public health policies and events using sentiment analysis. Sentiment analysis focuses on classifying and analyzing text sentiments. A Twitter sentiment analysis has the potential to monitor people’s attitudes towards public health policies and events. Here, we explore the feasibility of using Twitter data to build a surveillance system for monitoring people’s attitudes towards public health policies and events since the beginning of the COVID-19 pandemic. In this study, we conducted a sentiment analysis of Twitter data. We analyzed the relationship between the sentiment changes in COVID-19-related tweets and public health policies and events. Furthermore, to improve the performance of the early trained model, we developed a data preprocessing approach by using the pre-trained model and early Twitter data, which were available at the beginning of the pandemic. Our study identified a strong correlation between the sentiment changes in COVID-19-related Twitter data and public health policies and events. Additionally, the experimental results suggested that the data preprocessing approach improved the performance of the early trained model. This study verified the feasibility of developing a fast and low-human-effort surveillance system for monitoring people’s attitudes towards public health policies and events during a pandemic by analyzing Twitter data. Based on the pre-trained model and early Twitter data, we can quickly build a model for the surveillance system.

## 1. Introduction

### 1.1. Evidence-Based Health Policy

The importance of policy-relevant evidence for evaluating the potential impact of public health policies has been widely recognized [1,2,3]. An evidence-based public health policy (EBPH) aims to promote public health by fully utilizing available data for decision making [4]. Public health scientists have developed many approaches in this field [5,6,7,8]. These methods use evidence from multiple sources, such as medical literature, clinically gathered information, health information databases, and survey results. The success of applying EBPH strongly encourages researchers to advance analytical methods, e.g., health impact assessment, public health surveillance, and systematic reviews [2,9,10,11]. One common key component of these methods is data collection, which is still a challenging step [3]. Data collection is required to fit the time frame of the relevant and related policies [12,13]. Additionally, policy surveillance systems need to monitor patterns and trends of the related policy influence [14,15]. These systems require the data to be collected not only efficiently but also feasibly. Unfortunately, it is difficult to completely meet these requirements using traditional approaches, e.g., key informant interviews and case studies [3], due to the time delay caused by the inefficient processing steps of most of these approaches [16].

Recently, scientists attempted to apply big data in EBPH [17]. This study suggested that large-scale data might help with EBPH decision making. In the current era of the COVID-19 pandemic, there is an urgent need for fast and low-human-effort data collection approaches. To address these challenges, scientists have attempted to develop data collection approaches based on social media such as Facebook and Twitter [18]. The ease of accessing a large amount of real-time social media data gives social media unique advantages for EBPH [16,18,19,20,21]. The dynamic social media platforms enable scientists to study large populations [22]. Furthermore, the real-time nature of social media allows fast data processing to avoid the time delay caused by conventional approaches [16,23]. These features allow policymakers to collect real-time evidence easily. This advantage is crucial to EBPH, especially in the era of a pandemic.

Among these social media platforms, Twitter has been identified as a popular platform for short messages [19]. For instance, more than twenty percent of American adults are using Twitter [24]. Each Twitter message, called a tweet, is restricted to 280 characters. The setting meets the need for quick updates and makes Twitter more and more popular. As of the first quarter of 2020, Twitter, a microblogging web service, has 166 million daily active users [25], who send more than 500 million tweets every day [26]. The interactive nature of Twitter has attracted researchers to apply it to EBPH [19,27]. It is worth noting that the representativeness of the Twitter platform could be biased. A survey conducted by Pew Research Center in the United States indicated that the median age of adult Twitter users was 40, which was less than the median age of people residing in the United States [24]. Additionally, it was reported that the Twitter platform might incorrectly identify and filter out harmless Twitter messages [28].

### 1.2. Sentiment Analysis

Among the many tweet analysis applications in public health, tweet sentiment analysis has already received significant research attention. Sentiment analysis is a computational tool of natural language processing for studying the attitudes of the public on a topic [29,30]. Recently, machine learning approaches have made encouraging progress in sentiment analysis [31,32]. Twitter provides a huge volume of tweets, most of which are unstructured, public, short text messages. This enables applying sentiment analysis of tweets in multiple areas [33]. Some of these applications addressed public health issues. For example, researchers applied a Twitter sentiment analysis to investigate people’s attitudes towards public health concerns [34], the H1N1 pandemic [35], human papillomavirus (HPV) vaccines [36,37], drug-related issues [38], e-cigarettes [39], and diabetes [40]. These attitudes are critical to design related public health policies.

The recent COVID-19 pandemic strongly motivated researchers to apply Twitter sentiment analysis to related public health areas, such as the awareness of precautionary procedures for COVID-19 [41], social life impact of COVID-19 [42], concerns regarding COVID-19 [43], and emotional reactions towards COVID-19 [44,45,46]. To conduct the sentiment analysis, these studies either used pre-trained sentiment analysis models [45,46] or manually built annotated corpora for training the sentiment analysis models [41,42,43,44]. Both approaches have limitations. In comparison to using pre-trained models, training sentiment analysis models based on manually annotated corpora of a specific topic may achieve higher accuracy. However, the way people express their sentiments may dramatically vary during a pandemic. Therefore, the early annotated corpora and the early trained models could be outdated soon, although a pandemic may last for years. This disadvantage may lower the reliability of the early trained models. If we frequently conduct manual annotations for a surveillance system, it may significantly increase the required human efforts for this system and cause time delays.

### 1.3. Research Goals

The goal of this study was to explore the feasibility of using a fast and low-human-effort surveillance system for monitoring people’s attitudes towards public health policies and events during a pandemic such as COVID-19 by analyzing Twitter data. It is worth noting that evaluating people’s attitudes towards public health events can help us predict the people’s attitudes towards public health policies. Additionally, the study aimed to develop a computational approach for building a reliable model for the surveillance system based on the early Twitter data.

## 2. Materials and Methods

### 2.1. Data Preparation

On 28 February 2020, the WHO set the risk assessment of COVID-19 to “very high”. Since 1 March 2020, we used Tweepy [47], a free Python library for accessing Twitter APIs, to collect real-time tweets. From 1 March 2020 to 14 June 2020, we collected real-time tweets for hours at noon (US Eastern Time) every third day. Additionally, we collected related tweets at noon on 12 March due to the Europe travel ban announced by the United States government on March 11th. There were 37 data collection days. On each collection day, we received about 40,000 original tweets by searching the keywords “coronavirus”, “COVID-19”, “COVID19”, “COVID_19”, “SARSCOV2”, “SARS-COV-2”, and “SARS_COV_2”. These search keywords were from the official names of the disease and virus. To avoid confusion and unrelated tweets, this study did not use other names or terms as search keywords during tweet collection. Emojis and links in the collected tweets were removed by using a Python package [48], while slang was kept.

This study classified tweets into five sentiment levels: very negative, negative, neutral, positive, and very positive. Table 1 gives sample tweets representing the different sentiment levels. A big training data set would require much human effort to manually annotate all tweets of this data set. Therefore, we intended to verify whether a relatively small data set could still achieve good performance in this study. We randomly selected 700 tweets collected on March 1st. All 700 tweets and all words of a tweet were manually scored. Scores −2, −1, 0, 1, and 2 refer to very negative, negative, neutral, positive, and very positive, respectively. The annotated data set was called Mar01. Similarly, we randomly selected 2100 tweets collected from March to May and manually scored them. The annotated data set was called MarAprMay-all. This data set was separated into three data sets, each of which contained 700 annotated tweets. These three data sets were called MarAprMay-1, MarAprMay-2, and MarAprMay-3.

### 2.2. Sentiment Analysis Model Training

In this study, we used the Stanford CoreNLP toolkit [49] for sentiment classification. This is a pipeline framework for natural language processing (NLP). We used its recursive neural tensor network (RNTN) [50], a special type of recursive neural network (RNN), to conduct the sentiment analysis of tweets. The toolkit parses a preprocessed tweet into a binary tree. Each leaf node of this tree refers to a word. RNTN follows the bottom-up order to compute internal nodes by using a compositional function. Moreover, it classifies each node into one of the five sentiment categories: very negative, negative, neutral, positive, and very positive. Table 2 gives sample tweets representing different sentiment levels. These samples were from the corpus provided by the toolkit. The sentiment of the root node is that of the whole tweet. This toolkit contains a pretrained model, which was trained and tuned based on a large amount of existing annotated data, while it can also build new models by using user-specified training data sets. In our study, all training parameters were set by default. The number of training samples in a batch was 27. The training was allowed to repeat up to 400 iterations. The learning rate was 0.01. A cross-validation-like approach was used to train models. Each data set was randomly divided into seven subsets of equal size. For example, if the data set had 700 tweets, each subset had 100 tweets. One subset was used as the validation set, while the remaining subsets were assigned as training data. This process was repeated seven times. Each subset was assigned as the validation set once. Therefore, one data set had seven trained models. All seven models scored all tweets of the testing set. Each tweet’s final sentiment score was the average of seven scores. It is worth noting that our cross-validation-like approach was slightly different from the conventional cross-validation approach. The conventional cross-validation approach assigns one subset as the testing set instead of the validation set, while our approach has no impact on the testing set.

**Figure 1:**
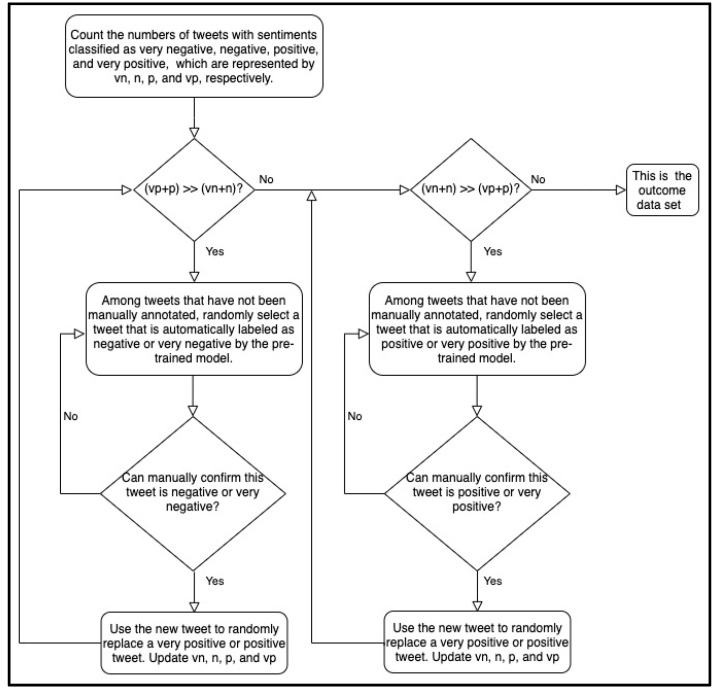
Brief pipeline of the preprocessing method with the close mode. The close mode requires the number of negative and very negative samples to be close to that of positive and very positive samples. Therefore, the resulting data set of the close mode is close to being balanced.

**Figure 2:**
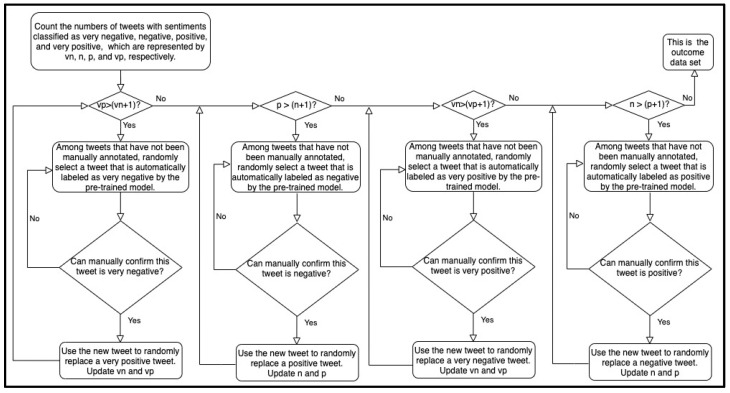
Brief pipeline of the preprocessing method with the complete mode. The complete mode requires the difference between the number of very negative samples and the number of very positive samples to be no more than one. This mode also requires the difference between the number of negative samples and the number of positive samples to be no more than one. Therefore, the resulting data set of the complete mode is completely balanced.

We used the data sets Mar01 and MarAprMay-all to build the early trained and late-trained models, respectively. The data set Mar01 was collected during the early stage of the pandemic, so it may have inherent biases due to the corpus incompletion. The training sample imbalance may significantly exacerbate the problem. Although most training algorithms do not require the numbers of positive and negative samples to be exactly equal, extremely unbalanced training data sets may worsen the training performance. Mar01 contained 366 negative or very negative samples and 122 positive or very positive samples. Here, we used the pretrained model to calibrate the training set of Mar01. We randomly selected tweets that were automatically labeled as positive or very positive by the pretrained model. After manually confirming that these tweets were positive or very positive, we added these tweets to the training set and removed the same number of negative or very negative tweets. We repeatedly updated the training set until it was balanced. Figure 1 and Figure 2 outline the brief preprocessing pipelines of balancing the data set with the close and complete modes, respectively. The close mode requires the number of negative and very negative samples to be close to that of positive and very positive samples. Therefore, the resulting data set of the close mode is close to being balanced. The complete mode requires the difference between the number of very negative samples and the number of very positive samples to be no more than one. This mode also requires the difference between the number of negative samples and the number of positive samples to be no more than one. Therefore, the resulting data set of the complete mode is completely balanced. However, the close mode requires less human effort than the complete mode does. Based on Mar01, the close mode generated a new data set, Mar01-Updated-1, while the complete mode generated the new data set Mar01-Updated-2. Table 3 gives the number of tweets in the five sentiment categories in each data set. At the early stage of the pandemic, the pretrained model and the models trained based on Mar01, Mar01-Updated-1, and Mar01-Updated-2 were available.

### 2.3. Sentiment Analysis

We used a model to assign all collected tweets a score of −2, −1, 0, 1, or 2, which refer to very negative, negative, neutral, positive, and very positive, respectively. The average score of a day was the overall sentiment score of that day. The method of computing the overall sentiment score for the i-th collection day, represented by $S_{i}$, is given as follows.

(1) $$S_{i}=\frac{Sum\left(sentimental\;scores\;of\;all\;tweets\;on\;day\;i\right)}{Number\;of\;tweets\;collected\;on\;day\;i},$$

where Sum() is the summation function, while i is an integer between 1 and 37. A high sentiment score suggests a positive attitude, while a low sentiment score indicates a negative attitude. The overall sentiment scores reflect the overall attitude of the population on a specific collection day. A surveillance system can analyze people’s attitudes during a pandemic by comparing overall sentiment scores on different collection days.

This study aimed to explore the feasibility of using a tweet sentiment analysis to build a surveillance system for monitoring people’s attitudes towards public health policies and events during a pandemic. The present investigation used late-trained models to compute the overall sentiment scores. We used the cross-validation-like approach, which is briefly introduced in Section 2.2, to train seven late-trained models based on the data set MarAprMay-all. The sentiment score of a tweet is the average score of seven scores. To investigate people’s attitudes towards specific public health policies and events, we needed to identify and analyze related tweets. This study conducted a sentiment analysis on three public health policies and events: the stay-at-home policy, the social distancing policy, and the mask wearing topic. Based on all collected tweets, we used “stay-at-home”, “stay at home”, “stay-home”, and “stay home” to identify tweets related to the stay-at-home policy. We also used “social distancing”, “social distance”, “socialdistancing”, “physical distancing”, “physical distance”, and “physicaldistancing” to identify tweets related to the social distancing policy. Similarly, we used “mask” to identify tweets related to the mask wearing topic based on all collected tweets. All of these were hot topics on Twitter for multiple collection days. It allowed us to collect enough tweets for analysis. In comparison, the Europe travel ban was only a one-day topic, which did not generate many tweets. These tweets were manually analyzed for identifying the reasons behind their sentiments. If more than ten percent of tweets on a specific topic were based on the same reason, we added this reason to the analysis results, which are given in Section 3.1.

### 2.4. Research Design

This study used the late-trained model to compute the overall sentiment scores of each collection day. To explore the feasibility of using Twitter data to build a surveillance system for monitoring people’s attitudes, we attempted to analyze significant sentiment changes in tweets related to public health events or policies. The analysis results will verify the feasibility of building a surveillance system based on Twitter data analysis.

We also tested the data preprocessing approach. At the beginning of the pandemic, there were three types of available models: the pretrained model, the early trained models based on the data set Mar01, and the early trained models based on the data sets Mar01-Updated-1 and Mar01-Updated-2. This work used two measurements to evaluate the performance of these models on the data sets MarAprMay-1, MarAprMay-2, and MarAprMay-3. One measure was average sentimental score error (*ASSE*), which is computed by the following expression:

(2) $$ASSE=\left|\frac{\sum_{i=1}^{n}Score_{i}^{manual}}{n}-\frac{\sum_{i=1}^{n}Score_{i}^{model}}{n}\right|,$$

where *n* is the total number of tweets in the data set, and $Score_{i}^{manual}$ and $Score_{i}^{model}$ refer to the sentiment scores of the *i-th* tweet based on manual annotation and model classification, respectively. A smaller *ASSE* suggests better performance. If the *ASSE* of a method is close to zero, it indicates that this method performs well with respect to the overall sentiment. The other measure was the mean squared error (*MSE*), which is calculated by the following expression:

(3) $$MSE=\frac{\sum_{k=1}^{n}{\left(Score_{k}^{manual}-Score_{k}^{model}\right)}^{2}}{n},$$

where *n* is the number of tweets in the data set, while $Score_{k}^{manual}$ and $Score_{k}^{model}$ refer to the scores of the *k-th* tweet based on manual annotation and model classification, respectively. *MSE* accumulates the errors of all sentiment scores. A smaller *MSE* indicates better performance.

## 3. Results

### 3.1. Sentiment Analysis Results

**Figure 3:**
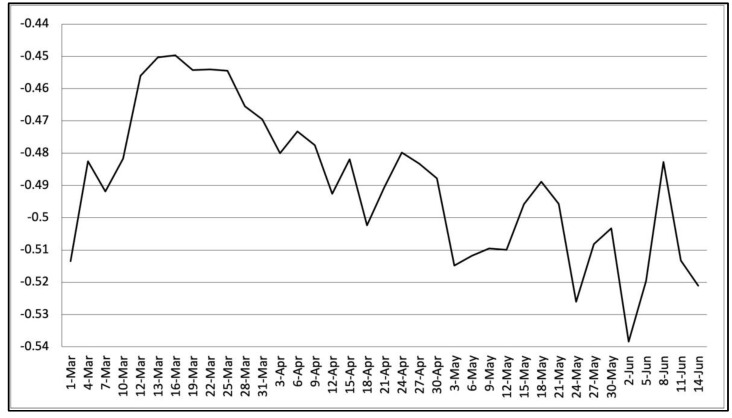
Overall sentiments of the population regarding COVID-19 from 1 March 2020, to 14 June 2020. The vertical axis indicates the overall sentiment score of each collection day, while the horizontal axis shows the collection dates.

The present investigation used the late-trained models to compute the overall sentiment scores, which are given in Figure 3. The rest of this section summarizes the analysis results of three public health policies and events: the stay-at-home policy, the social distancing policy, and the mask wearing topic. The analysis results provide critical information for public health policymakers for evaluating the potential impact of related policies.

#### 3.1.1. Analysis Results of the Stay-at-Home Policy

**Figure 4:**
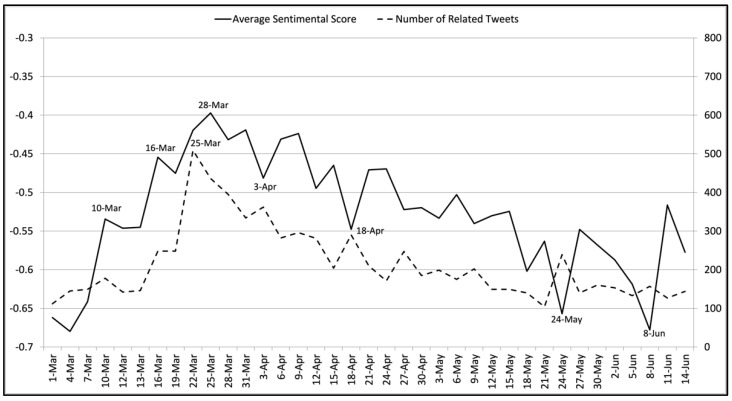
The average sentiment scores and the numbers of tweets related to the stay-at-home policy on each collection day from 1 March 2020 to 14 June 2020. The left vertical axis indicates average sentiment scores, while the right vertical axis suggests the number of related tweets collected. The horizontal axis shows the collection dates.

Figure 4 gives the average sentiment scores and the numbers of related tweets related to the stay-at-home policy. The sentiment scores and tweet numbers peaked on March 10th and 16th. A manual analysis showed that these peaks were strongly related to the stay-at-home suggestion given by the Centers for Disease Control and Prevention (CDC) of the United States for at-risk Americans on 10 March [51]. Most related tweets supported this suggestion. On 16 March, related tweets indicated that most people agreed with this suggestion. On 22 and 25 March, the tweet number and average sentimental score reached new peaks, respectively. Manual interpretation of the related tweets showed that these peaks reflected people’s attitudes towards the stay-at-home policy announced by more than 20 US states during this period. These tweets gave strong support to this policy. On 3 April, we observed a sentiment score valley and a tweet number peak. The related tweets reflected the frustration at job loss and COVID-19 virus spread, although the majority of tweets still showed support of the policy. A similar combination of sentiment score valley with tweet number peak was also observed on 18 April. The related tweets expressed concerns of the negative impacts of the policy. Figure 4 also shows combinations of sentiment score valleys and tweet number peaks on 24 May and 8 June. According to the related tweets, the former was triggered by the news regarding the British Prime Minister’s advisor breaking the stay-at-home policy [52], while the latter reflected people’s concerns of the COVID-19 virus spread after the stay-at-home policy was lifted.

#### 3.1.2. Analysis Results of the Social Distancing Policy

**Figure 5:**
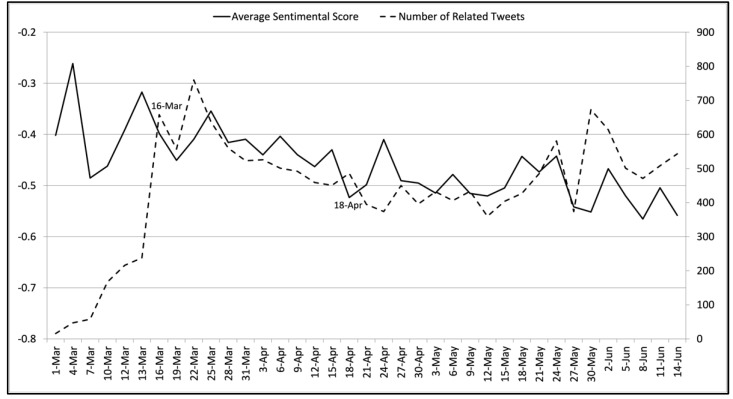
The average sentiment scores and the numbers of tweets related to the social distancing policy on each collection day from 1 March 2020 to 14 June 2020. The left vertical axis indicates average sentiment scores, while the right vertical axis suggests the number of related tweets collected. The horizontal axis shows the collection dates.

Figure 5 gives the average sentimental scores and the numbers of related tweets related to the social distancing policy. The then President of the United States suggested practicing social distancing on 16 March [53]. Related tweets collected on that day focused on the guideline of social distancing and gave support to this policy. We observed an average sentiment score valley with a tweet number peak on 18 April. Manual analysis of the related tweets suggested that it was caused by concerns of failing to keep social distance in the protests against the stay-at-home policy.

#### 3.1.3. Analysis Results of the Mask Wearing Policy

**Figure 6:**
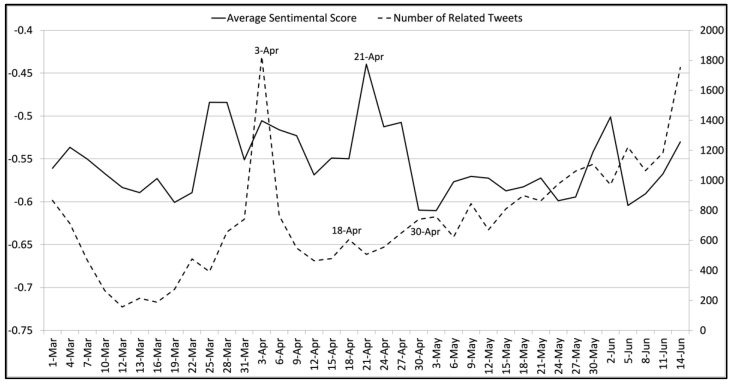
The average sentiment scores and the numbers of tweets related to the mask wearing topic on each collection day from 1 March 2020 to 14 June 2020. The left vertical axis indicates average sentiment scores, while the right vertical axis suggests the number of related tweets collected. The horizontal axis shows the collection dates.

Figure 6 gives the average sentiment scores and the numbers of tweets related to the mask wearing policy. On 3 April, the CDC recommended voluntarily wearing face masks in public [54]. Most related tweets claimed that using face masks would be helpful. This caused both the average sentiment score and the tweet number to peak on that day. We observed a sentiment score valley with a tweet number peak on April 18th. Manual analysis suggested that related tweets expressed concerns of people participating in protests without wearing face masks. Relatively low sentiment scores with a relatively high number of tweets were observed around 30 April. Most related tweets criticized Mike Pence for not wearing a face mask during his Mayo Clinic visit [55]. On 21 April, we observed a peak in average sentiment scores. Many related tweets showed enthusiasm for homemade face masks, which was inspired by related news [56].

### 3.2. Preprocessing Approach Evaluation

This section focuses on the performance comparison of the pretrained model, the early trained models without preprocessing, and the early trained models with preprocessing. ASSE and MSE were used to measure model performance. Lower ASSE and MSE values indicate better performance. The Stanford CoreNLP toolkit provided the pretrained model, which directly scored each tweet in the testing set. Other models were trained based on the cross-validation-like approach, which is briefly introduced in Section 2.2. The data set Mar01 was used to train seven early trained models without using the preprocessing method. The data set Mar01-Updated-1 was used to train seven early trained models with the close-mode preprocessing method. The data set Mar01-Updated-2 was used to train seven early trained models with the complete-mode preprocessing method. The score of a tweet with a specific type of model was the average score of seven related models.

Table 4 reports the experimental results. The models based on the data sets Mar01-Updated-1 and Mar01-Updated-2 outperformed the models based on the data set Mar01. This suggests that the proposed preprocessing method can consistently improve model performance. Furthermore, “Updated-Early-2” also outperformed the pretrained model in all tests, which indicates that the complete mode of the preprocessing method is reliable. “Updated-Early-1” also achieved a good overall performance and was the best in two tests. It shows that the close mode of the preprocessing method is useful, especially when the users have limited manpower. Additionally, “Updated-Early-1” outperformed “Updated-Early-2” in MSE on the testing set MarAprMay-1 and in ASSE on the testing set MarAprMay-3. It suggests that the training method of CoreNLP might overcome small gaps between the numbers of tweets with different sentiments.

## 4. Discussion

The findings of this study are in close agreement with previous studies [34,36,37,38,39] that applied sentiment analysis to analyze people’s attitudes regarding public health. Although the algorithm details of these studies are different, all of these studies demonstrated that sentiment scores could reflect people’s attitudes. Additionally, the overall sentiment scores were consistently low, which matches the expectation that the negative attitude of the overall population to the COVID-19 pandemic was strong from March 2020 to June 2020.

This study also explored the feasibility of building such a system at the beginning of a pandemic based on a pretrained model and early tweets. We proposed two data preprocessing modes, which are the close mode and the complete mode, for improving the performances of early trained models by balancing the training data set. Both modes achieved a significant performance improvement and outperformed the original early trained models. Both modes have shown an evident advantage in the computation of overall sentiment scores, which is evaluated by ASSE. This advantage meets the need of policy surveillance systems, which also focus on overall sentiment changes. The close mode is less labor-intensive, while the complete mode obtains higher overall accuracy. Therefore, if the surveillance system is very limited in manpower for data preprocessing, we recommend the close mode. Otherwise, the complete mode is suggested.

It is also worth noting that the current study did not conduct the sentiment analysis based on geographical and demographical information. For example, the current study cannot rule out the presence of tweets that may have been made by non-residents of a specific country, e.g., the United States, or a specific location of a country, e.g., Tennessee. As such, it did not investigate the sentiment changes towards the stay-at-home order in Tennessee. Our future work will address this issue by utilizing geographical and demographical information in sentiment analysis.

The present investigation focuses on the feasibility of building a fast and low-human-effort surveillance system for monitoring people’s attitudes towards public health policies and events during a pandemic such as the COVID-19 pandemic. This study demonstrates that it is feasible to build such a surveillance system by using tweet sentiment analysis. The tweet data are easy to collect, while tweet sentiment analysis can reveal the overall population’s sentiment changes. Recognizing these changes will assist public health policymakers in evaluating the potential impact of related policies. For example, if the surveillance system suggests that most people are strongly against some policies, policymakers may consider it as a reminder for policy adjustment. Utilizing geographical and demographical information in the surveillance system will enable the system to provide information regarding specific groups and help policymakers design policy details for addressing the specific needs of these groups. Our future work will tackle the geographical and demographical information issue for promoting the surveillance system.

## 5. Conclusions

The current investigation demonstrates that the overall sentiment score of tweet data may reflect attitude changes towards COVID-19. It suggests the feasibility of building a fast and low-human-effort surveillance system for monitoring people’s attitudes towards public health policies and events during a pandemic such as COVID-19. This study investigated attitude changes on some public health topics. For example, the tweet data analysis showed that many people changed their attitudes towards the stay-at-home policy due to the negative impacts of this policy. Furthermore, we also explored the use of a pretrained model and early tweets for this surveillance system. In our future work, we will focus on enhancing our approach by improving the early trained model and addressing the geographical and demographical information issue.

## Figures and Tables

**Table 1:** Sample tweets representing different sentiment levels based on manual annotation.

Level	Sample Tweet
Very negative	The U.S. response to the coronavirus pandemic (it is that, just not acknowledged yet) is *shameful*. For the richest country in the world to fumble this badly is inexcusable. #COVID19 #coronavirus #pandemic
Negative	#coronavirus again exposing how there is no civilisation anywhere in the world.
Neutral	If you ever went to a party at the greenhouse, you’re immune to the coronavirus
Positive	Maybe the Masked Singer had the right idea #SocialDistancing #Isolation #COVID19 #Coronavirus
Very positive	#Erie County (275k pop.): 3.6% #COVID19 positive test rate (1054 tests, 38 positives w/.38% of pop. tested). PA (12M pop.): 18% positive test rate (120,153 tests, 21,655 pos. w/1% of pop. tested). Fewer tested, yes. But especially happy to be an Erie County resident right now.

**Table 2:** Sample tweets representing different sentiment levels based on the pretrained model. These samples were from the corpus provided by the Stanford CoreNLP toolkit [49].

Level	Sample Tweet
Very negative	A dull, simple-minded and stereotypical tale of drugs, death and mind-numbing indifference on the inner-city streets.
Negative	A sleek advert for youthful anomie that never quite equals the sum of its pretensions.
Neutral	So here it is: It’s about a family of sour immortals.
Positive	Any movie that makes hard work seem heroic deserves a look.
Very positive	A good music documentary, probably one of the best since The Last Waltz.

**Table 3:** The numbers of tweets in the five sentiment categories in each data set.

Data Set	Very Negative	Negative	Neutral	Positive	Very Positive
Mar01	35	331	212	115	7
Mar01-Updated-1	26	237	212	201	24
Mar01-Updated-2	21	223	212	223	21
MarAprMay-1	37	296	233	121	13
MarAprMay-2	19	271	269	128	13
MarAprMay-3	17	259	277	132	15
MarAprMay-all	73	826	779	381	41

**Table 4:** Performance comparison on the testing data sets MarAprMay-1, MarAprMay-2, and MarAprMay-3. Smaller ASSE and MSE indicate better performance. “Pretrained” refers to the pretrained model. “Original early trained” refers to the early trained models trained by the data set Mar01. “Updated-Early-1” refers to the early trained models trained by the data set Mar01-Updated-1. “Updated-Early-2” refers to the early trained models trained by the data set Mar01-Updated-2.

Method	MarAprMay-1		MarAprMay-2		MarAprMay-3	
	ASSE	MSE	ASSE	MSE	ASSE	MSE
Pretrained	0.346	0.783	0.283	0.731	0.293	0.767
Original early trained	0.156	0.810	0.106	0.769	0.152	0.781
Updated-Early-1	0.036	0.699	0.052	0.753	0.025	0.707
Updated-Early-2	0.030	0.721	0.037	0.672	0.026	0.645

## Data Availability

The data presented in this study are openly available in Github at https://github.com/mengtsai1988/COVID-19_TweetsRawData, accessed on 9 June 2021.
